# Complex N acquisition by soil diazotrophs: how the ability to release exoenzymes affects N fixation by terrestrial free-living diazotrophs

**DOI:** 10.1038/ismej.2016.127

**Published:** 2016-11-29

**Authors:** Jeffrey S Norman, Maren L Friesen

**Affiliations:** 1 Department of Plant Biology, Michigan State University, East Lansing, MI, USA

## Abstract

Terrestrial systems support a variety of free-living soil diazotrophs, which can fix nitrogen (N) outside of plant associations. However, owing to the metabolic costs associated with N fixation, free-living soil diazotrophs likely rely on soil N to satisfy the majority of cellular N demand and only fix atmospheric N under certain conditions. Culture-based studies and genomic data show that many free-living soil diazotrophs can access high-molecular weight organic soil N by releasing N-acquiring enzymes such as proteases and chitinases into the extracellular environment. Here, we formally propose a N acquisition strategy used by free-living diazotrophs that accounts for high-molecular weight N acquisition through exoenzyme release by these organisms. We call this the ‘LAH N-acquisition strategy' for the preferred order of N pools used once inorganic soil N is limiting: (1) low-molecular weight organic N, (2) atmospheric N and (3) high-molecular weight organic N. In this framework, free-living diazotrophs primarily use biological N fixation (BNF) as a short-term N acquisition strategy to offset the cellular N lost in exoenzyme excretion as low-molecular weight N becomes limiting. By accounting for exoenzyme release by free-living diazotrophs within a cost–benefit framework, investigation of the LAH N acquisition strategy will contribute to a process-level understanding of BNF in soil environments.

## Introduction

Biological nitrogen fixation (BNF), the ability to reduce atmospheric N_2_ gas to NH_3_, is a process performed by a diverse array of prokaryotic microorganisms collectively known as diazotrophs. Although many diazotrophs, including those from genera such as *Rhizobia* and *Frankia*, fix nitrogen (N) in symbiotic associations with plants, soils also contain large numbers of free-living diazotrophs, which are responsible for a high proportion of BNF in many terrestrial environments (Cleveland *et al.*, 1999; Reed *et al.*, 2011). However, free-living soil diazotrophs have access to multiple fixed N sources and they likely use N acquisition strategies that take advantage of both atmospheric N and soil N pools. Here we explore the N acquisition strategies used by free-living diazotrophs with a particular focus on how the presence of high-molecular weight organic N sources, which must be degraded outside the cell, affect rates of BNF by these organisms. We then outline a strategy by which these organisms may acquire N (the ‘LAH' N acquisition strategy), and investigate the potential ecosystem-level ramifications of this strategy on BNF by free-living diazotrophs.

## N sources available to free-living diazotrophs in soils

By definition, free-living soil diazotrophs have access to N_2_ gas (hereafter atmospheric N) through BNF, but soils contain a variety of fixed N compounds (hereafter soil N) that can support their growth as well. We divide soil N into three categories: inorganic N, low-molecular weight organic (LMW) N and high-molecular weight organic (HMW) N. Rather than assigning specific size limits on LMW N and HMW N, we prefer to take a functional approach to soil N categorization based on how organisms can access these pools: LMW N are forms of organic N small enough for direct uptake by microbes, whereas HMW N are forms of organic N too large for direct uptake; HMW N must therefore be degraded in the extracellular environment before access by soil microbes. We expand on these categories below.

Inorganic soil N includes NH_4_^+^/NH_3_ and NO_3_^−^; whereas soil processes create and consume NO_2_^−^ as well, this resource rarely accumulates to levels comparable to those of NH_4_^+^/NH_3_ or NO_3_^−^ in natural systems (Van Cleemput and Samatar, 1996) and we therefore avoid further discussion of soil NO_2_^−^. LMW N includes organic monomers of N, such as amino acids and N-containing sugars (for example, *N*-acetyl glucosamine, the building block of chitin) and short chain polymers such as N-containing oligosaccharides and oligopeptides, which may be preferentially accessed through direct uptake by soil microbes on a global scale (Farrell *et al.*, 2013). HMW N sources in soil include organic N-containing biopolymers, such as proteins or chitin, and highly complex organic molecules that either occlude inorganic N or contain N in ring structures, (for example, heteocyclic N, which is formed in fire-affected systems; Knicker, 2011). N-acquiring exoenzymes, which many microbes release to access HMW N in soil environments, therefore include both enzymes that degrade N-containing polymers, such as proteases and chitinases, and oxidative enzymes that degrade complex soil organic matter, such as peroxidases and phenol oxidases (Sinsabaugh, 2010).

### HMW N acquisition is common among free-living soil diazotrophs

Although many soil microbes are able to acquire HMW N through the release of N-acquiring exoenzymes (Geisseler *et al.*, 2010 and references therein), we wanted to investigate how common this ability was in free-living soil diazotrophs. Here we present two independent lines of evidence suggesting that a variety of free-living soil diazotrophs have access to the HMW N pool in soil. First, we summarize reports of exoenzyme production by free-living soil diazotrophs in pure culture (Table 1). Second, we show genomic evidence for exoenzyme production by free-living soil diazotrophs (Table 2).

To search for reports of exoenzyme production by free-living soil diazotrophs in pure culture, we took advantage of the fact that organisms are often screened for the ability to produce proteases and chitinases as a part of routine strain characterization when new microbial species are described. We therefore conducted a literature search of recently introduced species of free-living soil diazotrophs (that is, those reported since the year 2000) and found evidence for the ability to degrade HMW N in multiple genera from the *Proteobacteria*, *Bacteroidetes* and *Firmicutes* isolated from a variety of soil environments (Table 1). However, the substrates tested, particularly the animal-derived proteins casein and gelatin, may not be highly abundant in soil systems, and no oxidative enzymes are typically included in this nature of testing. We therefore supplemented these findings by searching for genomic evidence of exoenzyme production in five additional free-living soil diazotrophs.

To obtain genomic evidence of exoenzyme production by free-living soil diazotrophs, we downloaded all annotated protein sequences from five known diazotrophic organisms with fully sequenced genomes from the NCBI genome database (www.ncbi.nlm.nih.gov/genome; accessed on 1/6/2016), then screened sequences for signal peptides, which mark them for excretion, using the SignalP 4.1 server (www.cbs.dtu.dk/services/SignalP; Peterson *et al.*, 2011). Annotations of proteins that tested positive for signal peptides were inspected to determine whether they qualified as N-acquiring exoenzymes. These sequences were then aligned against the non-redundant protein database using BLAST (www.blast.ncbi.nlm.nih.gov) to ensure that they were not closely related to enzymes of other functions (for example, enzymes involved in sporulation). We limited our search to organisms that were first isolated from soils, which are not known to form symbiotic associations with plants and for which BNF has been verified in pure culture. Methodological limitations constrained our search to Gram-positive bacteria as SignalP cannot differentiate between exoenzymes and periplasmic enzymes in Gram-negative organisms, and SignalP is not capable of detecting signal peptides in Archaea (Peterson *et al.*, 2011). Using these methods, we found genomic evidence for the ability to produce N-acquiring exoenzymes in all five organisms tested (Table 2). It should be noted that the SignalP pipeline does a poor job of identifying twin-arginine translocation signal peptides, an alternate class of signal peptides used by bacteria, likely leading us to underestimate the abundance of N-acquiring exoenzymes in the genomes tested (Peterson *et al.*, 2011).

Overall, the results from two independent surveys suggest that the ability to acquire HMW N is both phylogenetically and geographically widespread among free-living soil diazotrophs. Most of the N-acquiring exoenzymes we detected are proteases, which is unsurprising as approximately half of the dissolved organic N pool in soils is thought to be composed of hydrolysable amino acids (that is, peptides including proteins; Warren, 2014). We found no evidence for oxidative exoenzyme production in the diazotrophic genomes we investigated.

Although our search for exoenzymes was intended to find evidence for extracellular degradation of organic N polymers by free-living diazotrophs, we also found evidence for production of extracellular amine oxidase enzymes by all three *Paenibacillus* species, but neither *Clostridium* species that we investigated (Supplementary Table 1). Extracellular deamination enzymes liberate NH_3_ from LMW N sources, making it available for direct uptake. Although we cannot tell the substrate specificity of the enzymes we detected, it seems likely that most are extracellular amino-acid oxidases as soil microbes are known to express these enzymes (Geisseler *et al.*, 2010 and references therein) and amino acids comprise up to 88% of the dissolved monomeric LMW N pool in soils (Warren, 2014 and references therein).

Extracellular amino-acid oxidases are adhered to the outside of the cell in the bacteria (Böhmer *et al.*, 1989; Braun *et al.*, 1992) and fungi (Davis *et al.*, 2005; Nuutinen and Timonen, 2008) that express them; enzymes adhered to the cell surface are excreted from the cell and would therefore be detected by the SignalP server. We discuss the consequences that the production of cell surface-adhered amino-acid oxidases may have for BNF by free-living soil diazotrophs later; it should be noted that these enzymes are not included in our definition of N-acquiring exoenzmyes, a term we reserve for enzymes with HMW N substrates.

## A cost–benefit analysis of N acquisition by free-living soil diazotrophs

Access to inorganic, LMW, HMW and atmospheric N requires different cellular N investments (for example, protein construction) and energetic investments (for example, ATP) by free-living soil diazotrophs. Furthermore, the return on these investments differs for each N pool. Understanding both the investments that diazotrophs must make to access available N pools, as well as the potential returns on these investments, is necessary to predict the N acquisition strategies of these organisms. Here we outline the cellular N and energetic investments free-living soil diazotrophs must make to access inorganic, LMW, atmospheric, and HMW N; we also discuss the potential returns on each of these investments. This information is summarized in Table 3.

### Inorganic N acquisition

Free-living soil diazotrophs access inorganic N through direct uptake. Cellular N investments required for inorganic N acquisition by free-living soil diazotrophs vary with inorganic N availability but are low in comparison with other forms of N acquisition. As an uncharged small molecule, NH_3_ can penetrate cell membranes through passive diffusion, and many microbes therefore do not construct NH_3_/NH_4_^+^ transport proteins when NH_3_ is highly abundant (Kleiner, 1981), as may be the case in high NH_4_^+^ and/or alkaline soils. In cases of lower NH_3_ availability, cellular N investments are required to construct proteins for NH_3_/NH_4_^+^ uptake either through facilitated diffusion or active transport. Proteins must be constructed for NO_3_^−^ uptake regardless of availability and cellular N resources must be diverted toward proteins involved in NO_3_^−^ reduction as well.

Energetic investments necessary to acquire inorganic N vary from low to moderate in comparison with other N acquisition strategies; these investments vary because of environmental concentration and the oxidation state of inorganic N. Energetic investments increase to acquire inorganic N at low availability, as microbes must switch to using active transport mechanisms (Kleiner, 1993) and/or high-affinity NH_3_ assimilation pathways that require ATP investment (Reitzer, 2003). NH_4_^+^ is incorporated into biomass with little energetic expenditure, whereas 1 molecule of ATP and 8 reducing equivalents, which can be derived from ~1/3 molecule of glucose using aerobic respiration (Groβkopf and LaRoche, 2012), are necessary to reduce NO_3_^−^ before incorporation into biomass.

The energetic investments required for NO_3_^−^ assimilation are further complicated by the role of NO_3_^−^ as a terminal electron acceptor for anaerobic respiration. As pairing organic carbon oxidation with NO_3_^−^ reduction (that is, anaerobic respiration) allows microorganisms to maximize the energetic returns from respiration in anaerobic environments, there exists an energetic opportunity cost for using NO_3_^−^ to satisfy cellular N demand, rather than respiratory demand, in oxygen-limited conditions. The fact that high energetic yields can be gained from NO_3_^−^ respiration could make NO_3_^−^ unavailable for use as a cellular N source in anaerobic conditions, effectively excluding NO_3_^−^ from the inorganic N pool when oxygen is absent.

The returns on investments necessary to acquire inorganic N are immediate when this resource is available. However, these returns are ultimately governed by inorganic N availability, which can be quite low in comparison with organic N availability in soil (Geisseler *et al.*, 2010; Warren, 2014 and references therein).

### LMW N acquisition

Free-living soil diazotrophs access LMW N through direct uptake and extracellular deamination. We do not know how phylogenetically widespread extracellular deamination is among free-living soil diazotrophs since we believe that this is the first published evidence that this functional group has the the ability to produce these enzymes. The fact that we found evidence for extracellular deamination in all investigated *Paenibacillus* species, but no *Clostridium* species, suggests that this ability may cluster phylogenetically. We discuss the processes of LMW N uptake and extracellular deamination separately.

Although the acquisition processes are mechanistically similar, the cellular N investments necessary for LMW N uptake may slightly exceed those necessary for inorganic N uptake. This is because a larger variety of transporters are required for LMW N uptake than inorganic N uptake, and transporter proteins must be expressed regardless of LMW N availability as LMW N cannot diffuse through cellular membranes. Energetic investments necessary for LMW N uptake include those required to construct transport proteins, those required to fuel active transport for LMW N sources common inside cells such as amino acids (Aranku, 1980) and those required to convert LMW N into usable forms. There may be tradeoffs in energetic investments required for uptake and use of LMW N resources: for example, while many amino acids can be used by diazotrophic cells to fulfill cellular N demand without alteration, access by uptake requires active transport; conversely LMW N sources that are not common components of diazotrophic cells may be brought in by facilitated diffusion but require modification before use. Interestingly, Matthews and Payne (1980) suggested that energetic investments necessary for uptake and use of oligopeptides may be lower than those necessary for use of other LMW N resources. This is because cells hydrolyze oligopeptides into constituent amino acids, which can satisfy cellular N demand with little or no modification, once they enter the cell; immediate hydrolysis keeps concentrations of oligopeptides inside cells low, thereby allowing for continued uptake by facilitated diffusion rather than active transport.

As we have seen, some free-living soil diazotrophs express surface-bound deamination enzymes as a means of LMW N acquisition. This process is functionally similar to LMW N uptake as deamination occurs at the cell surface, ensuring that the cells that produce these enzymes directly benefit from their activity. Production of extracellular deamination enzymes likely requires similar cellular N and energetic investments as the production of LMW N uptake channels. NH_3_ uptake channels are necessary to capture the NH_3_ liberated by deamination enzymes, but they do not require additional investment as they are constantly expressed except in cases of extremely high NH_3_ availability (Geisseler *et al.*, 2010). There may be an energetic opportunity cost to accessing LMW N through extracellular, rather than intracellular, deamination if cells do not uptake and metabolize carbon-rich byproducts of deamination. However, this fact is countered by the observation that amino-acid oxidase enzymes produce the toxic byproduct H_2_O_2_, which must be dealt with if deamination occurs in the intracellular environment (Geisseler *et al.*, 2010). Overall, LMW N uptake and extracellular deamination likely require similar investments and yield similar returns, and we therefore treat LMW N acquisition as a singular process from a cost–benefit perspective (Table 3).

The returns on the investments necessary to acquire LMW N are immediate when LMW N is available. However, much like inorganic N, these returns are ultimately governed by the availability of this resource.

### Atmospheric N acquisition

Atmospheric N acquisition by BNF requires moderate cellular N investment; nitrogenase proteins, which drive BNF, make up as much as 10% of total cellular protein during BNF (Dingler *et al.*, 1988), and construction of these N-rich molecules therefore requires a substantial up front investment of cellular N by free-living soil diazotrophs. This investment must be met by uptake of environmental N, and not by reallocation of cellular N, as demonstrated by the fact that many diazotrophs require a minimum level of initial fixed N to fuel diazotrophic growth in pure culture (Jensen and Holm, 1975). However, BNF can supply the cellular N for continued nitrogenase production once this initial N investment is satisfied (Hill, 1992).

BNF requires moderate to high energetic investment by free-living soil diazotrophs; variations in investments required are largely driven by the oxygen sensitivity of nitrogenase proteins, which are irreversibly destroyed by oxygen. BNF requires direct energetic investment to overcome the high activation energy necessary to break the triple bonded N_2_ molecule and to reduce it: overall, 16 molecules of ATP and 8 reducing equivalents are required to reduce one molecule of N_2_ gas (Hill, 1992). This direct cost of BNF is actually comparable to that of NO_3_^−^ reduction when driven by aerobic respiration in that ~1/3 glucose molecules can produce the ATP and reducing equivalents directly required to produce 1 molecule of NH_3_ by BNF (Groβkopf and LaRoche, 2012).

However, the energetic costs of BNF greatly increase in aerobic conditions, where free-living soil diazotrophs must invest substantial energetic resources into oxygen protection of nitrogenase proteins through increased respiration and exopolysaccharide production to slow oxygen diffusion into cells (Hill, 1992). For these reasons, *Azotobacter vinelandii,* a common aerobic free-living soil diazotroph, has been shown to require 100 g of glucose to fix 1 g of N (~7.7 molecules of glucose per molecule of N) in pure culture (Gutschick, 1982). Similar estimates show that anaerobic diazotrophs require between 8 and 30 molecules of glucose per molecule of N fixed (Gutschick, 1981), suggesting that costs of oxygen protection are ameliorated by increased energy yields associated with aerobic respiration. BNF can be accomplished for the least energetic cost in microaerophilic conditions, where diazotrophs benefit from the increased energetic yields associated with aerobic respiration while investing less energy on oxygen protection of nitrogenase enzymes.

There are guaranteed immediate returns on investments toward BNF as atmospheric N is likely never limiting. Energetic investments in BNF are necessarily continuous as both ATP and reducing equivalents must be applied for continued fixation.

### HMW N acquisition by exoenzyme production

Free-living diazotrophs access HMW N through the secretion of N-acquiring exoenzymes. Exoenzyme production requires high cellular N investment and moderate energetic investment as outlined below (see Burns *et al.* (2013) for a more extensive review of the investments required for exoenzyme production). The cellular N investments necessary for HMW N acquisition are high in comparison with other N sources as HMW N acquisition necessitates the excretion of N-rich molecules (that is, N-acquiring exoenzymes) from the cell. Energetic investments are required for both exoenzyme synthesis and excretion as well. Interestingly, Smith and Chapman (2010) showed that ATP costs of exoenzyme synthesis are lower than those for non-excreted proteins in *Escherichia coli*; this finding could extend to free-living diazotrophs as well. There is an energetic opportunity cost to exoenzyme excretion as the carbon lost when these molecules are excreted from the cell is not available for energy production.

There are no guaranteed returns on the investments required for HMW N acquisition as the LMW N created by the activity of N-acquiring exoenzymes may be available to microbes other than those directly involved in their production. However, microbes use cell-to-cell signaling pathways to coordinate group participation in exoenzyme production (Burns *et al.*, 2013) leading to a higher likelihood of return on this investment; quorum sensing pathways also enhance potential returns by serving as a proxy for the diffusion environment. Much HMW N in soil may be physically protected from enzymatic degradation because of interaction with the soil matrix (Cotrufo *et al.*, 2013), which likely decreases returns on investments for exoenzyme production in some environments. Returns on investments are also not immediate because of a temporal delay between exoenzyme secretion and increased LMW N availability. However, microbes do not need to continuously invest in exoenzyme production as N-acquiring enzymes persist in the environment because of stabilization by soil particles (Wallenstein and Weintraub, 2008). Indeed, some studies suggest that exoenzyme activity can remain stable for years after release (for example, Olander and Vitousek, 2000).

## Free-living diazotrophs are predicted to follow a LMW N, atmospheric N, HMW N (LAH) strategy for N acquisition when inorganic N is limiting

Free-living soil diazotrophs are routinely exposed to inorganic, LMW, HMW and atmospheric N in combination in their native environments. When inorganic N is available, free-living diazotrophs should preferentially use this resource over both soil organic N and atmospheric N because of the low energetic and cellular N investments necessary for inorganic N uptake, and the high probability of returns on these investments. Indeed, ample pure culture evidence is available to show that free-living diazotrophs preferentially use NH_3_/NH_4_^+^ over atmospheric N (Gutschick, 1981; Hill, 1992; Reed *et al.*, 2011; and references therein). Assimilatory NO_3_^−^ reduction is similarly favored over BNF by many diazotrophs as well; for example, NO_3_^−^ represses BNF by both *Azotobacter vinelandii* (Wilson *et al.*, 1943) and *Azosopirillum brasilense* (Gallori and Bazzicalupo, 1985), two commonly studied free-living soil diazotrophs.

Some free-living soil diazotrophs use NO_3_^−^ for anaerobic respiration and these organisms have evolved N acquisition strategies that do not prioritize NO_3_^−^ as a source of cellular N. For example, BNF by *Azospirillum lipoferrum* is not repressed by the addition of NO_3_^−^ to the medium, and this organism will actually use atmospheric N to fill cellular N demand, while using NO_3_^−^ as a terminal electron acceptor for anaerobic respiration (Neyra and Van Berkum, 1977); this seemingly bizarre behavior underscores the energetic importance of NO_3_^−^ as a terminal electron acceptor in anaerobic conditions. If diazotrophs are not using NO_3_^−^ for cellular N, this resource is functionally excluded from the inorganic N pool for these organisms.

Free-living soil diazotrophs should prioritize inorganic N uptake over both LMW and HMW N acquisition as well because of the higher cellular N and energetic investments required to access these pools. Indeed, pure culture studies routinely show repression of amino-acid transport in the presence of 10–25 μM of NH_4_^+^ (Geisseler *et al.*, 2010 and references therein) and high levels of NH_4_^+^ have been shown to repress protease activity (Allison and Macfarlane, 1992) and chitinase activity (Bidochka and Khachourians, 1993) as well. Furthermore, Allison and Macfarlane (1992) showed that protease activity was repressed when soils were amended with high levels of NH_4_^+^.

**Figure 1:**
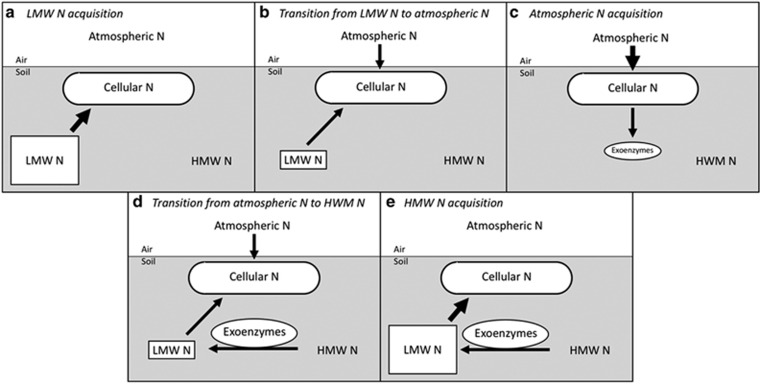
Conceptual outline of the LAH N acquisition strategy. Box size represents pool size and arrow thickness represents flux rate. (**a**) LMW N acquisition. Free-living soil diazotrophs preferentially access high-quality LMW N through direct uptake when inorganic N is exhausted. (**b**) Transition from LMW N in atmospheric N acquisition. As LMW N availability decreases, free-living soil diazotrophs simultaneously access remaining LMW N and atmospheric N to fulfill cellular N demand. (**c**) Atmospheric N acquisition. When LMW N is exhausted, free-living soil diazotrophs rely on atmospheric N to satisfy cellular N demand and obtain N for the production of N-acquiring exoenzymes. (**d**) Transition from atmospheric N to HMW N. As exoenzymes accumulate in the extracellular environment, their activity leads to increased LMW N availability, which in turn represses exoenzyme production. Increased LMW N availability also decreases diazotrophic reliance on BNF. (**e**) HMW N acquisition. Exoenzymes continue to regenerate LMW N pools through HMW N degradation without continued investment by diazotrophs. High LMW N availability causes free-living soil diazotrophs to cease reliance on BNF as well.

However, we seek to understand the behavior of these organisms when inorganic N is limiting, as may often be the case in natural systems; understanding how free-living soil diazotophs access organic N supplies available in soils is key to understanding process-level BNF in terrestrial environments. When inorganic N is unavailable, we propose that free-living diazotrophs access N pools in the following order: (1) LMW N, (2) atmospheric N, and (3) HMW N. We have named this the ‘LAH N-acquisition strategy', an abbreviation that refers to the order in which N pools are accessed. A conceptual diagram of the LAH N acquisition strategy is presented in Figure 1. We note that while organic N sources could be used to fill energetic demand by diazotrophs as well as cellular N demand, we focus on the latter since our goal is to predict the N acquisition strategies, rather than carbon acquisition strategies, used by free-living diazotrophs.

### Introduction of the LAH N acquisition strategy

Owing to the low energetic and cellular N investments necessary to acquire LMW N (Table 3), free-living soil diazotrophs would benefit from preferentially using this resource over both atmospheric N and HMW N when inorganic N is unavailable. LMW N is preferentially used over HMW N by non-diazotrophic microbes in pure culture, primarily due to end-product repression of exoenzyme synthesis. For example, high levels of amino acids have been shown to repress extracellular protease production in a variety of strains (Glenn, 1976 and references therein) and high levels of *N*-acetyl-glucosamine have been shown to repress chitinase production in pure culture as well (Felse and Panda, 1999 and references therein). Free-living soil diazotrophs should similarly repress the synthesis of N-acquiring exoenzymes when LMW N is abundant in soils (Figure 1a).

Although higher energetic and cellular N investments are required for BNF than LMW N uptake (Table 3), high LMW N availability does not always completely repress BNF by free-living diazotrophs in pure culture. For example, although some amino acids do completely repress BNF by particular diazotrophs, other amino acids only partially repress BNF, and the patterns of amino-acid use may vary between closely related taxa (references for this behavior are provided below). Although the immediate benefits of incomplete repression of BNF by free-living soil diazotrophs in the presence of LMW N are not apparent, the competitive advantages of this behavior become clear when the cellular N costs of exoenzyme production are taken into account, as we shall see later. First, we discuss pure culture evidence for this phenomenon and explore the role of LMW N in regulation of BNF in soils.

Hartmann *et al.* (1988) tested the effects of amino-acid addition on BNF by four different species of the genus *Azospirillum*. Although BNF (measured by acetylene reduction) by several strains of *A. lipoferrum* was almost completely inhibited by addition of the four amino acids tested, BNF by *A. brasilense* strains showed little or no repression by serine and histidine, partial repression by glutamate, and nearly full repression by alanine. Interestingly, extracellular deamination may provide a possible mechanism by which some amino acids completely inhibit fixation because this process leads to elevated concentrations of NH_3_ in the vicinity of the cell surface. Furthermore, diauxic growth patterns such as those exhibited when NH_3_ is added to the medium are not observed when diazotrophs are exposed to certain amino acids in pure culture, and diazotrophs balance BNF and LMW N uptake to maintain consistent growth even as LMW N availability decreases (Gadkari and Stolp, 1976).

Although the aforementioned experiments have focused on the behavior of free-living soil diazotrophs in the presence of single LMW N compounds, these organisms are simultaneously exposed to a mixture of LMW N sources in soils. Some of these LMW N sources will fully repress fixation by particular free-living soil diazotrophs at high enough availability, while others may not repress BNF at all. Although the concentration of LMW N compounds that fully repress BNF is likely high enough to do so when free-living soil diazotrophs first experience inorganic N limitation (Figure 1a), the concentration of these individual compounds within the diverse LMW N pool in soils will fall below repression levels as these organisms access LMW N; this may happen quickly if these compounds are preferentially used by free-living diazotrophs. Overall, free-living diazotrophs will begin to supplement LMW N acquisition with BNF to meet cellular N demand as LMW N availability decreases (Figure 1b).

Low LMW N availability has been shown to induce production of N-acquiring exoenzymes by non-diazotrophic soil microbes (Geisseler *et al.*, 2010 and references therein) and exoenzyme production by free-living diazotrophs is likely induced by low LMW N availability as well. As discussed previously, acquisition of HMW N comes at a substantially higher cellular N cost than other forms of N acquisition (Table 3) and non-diazotrophic microbes must use cellular N acquired through LMW N uptake to meet this cost. However, free-living diazotrophs use BNF to supplement cellular N resources, while LMW N is still available; although this behavior does not seem to make sense from a cost–benefit perspective (Table 3), increased diazotrophic reliance on BNF, while LMW N is still available ensures that free-living diazotrophs have ample cellular N available for exoenzyme production when LMW N runs out. Free-living soil diazotrophs can therefore fully exhaust the LMW N pool before releasing N-acquiring exoenzymes without losing the ability to meet cellular N investments necessary for exoenzyme production (Figure 1c).

As N-acquiring exoenzymes accumulate in the extracellular environment, LMW N availability will increase, once again inhibiting exoenzyme production and decreasing reliance on atmospheric N by free-living soil diazotrophs (Figure 1d). BNF may be entirely inhibited as LMW N availability increases further due to the likelihood that concentrations of LMW N sources that fully repress fixation will once more increase above repressing concentrations. BNF may also be inhibited if inorganic N pools are regenerated through abiotic import or through the activity by other microbes.

### Competitive advantages of the LAH N acquisition strategy

Free-living soil diazotrophs with the ability to release N-acquiring exoenzymes can access all three soil N pools available to non-diazotrophic microbes in addition to atmospheric N. As the cellular N costs of BNF are far lower than those of HMW N acquisition (Table 3), it would seem that free-living diazotrophs would gain a competitive advantage by avoiding competition for HMW N and relying on atmospheric N to satisfy cellular N demand once inorganic N and LMW N resources are exhausted. However, this would be a poor long-term N acquisition strategy as continuous energetic investment is required to obtain atmospheric N through BNF. Exoenzyme production is a better long-term N acquisition strategy as exoenzmyes can become stabilized by soil particles and continue to degrade HMW N without continuous investment (Table 3). However, exoenzyme production is a poor short-term N acquisition strategy as it requires substantial cellular N investment up front and there is a lag between the start of exoenzyme production and the return on this investment. Although the short-term failings of exoenzyme release adversely affect non-diazotrophs, free-living diazotrophs can use the LAH N acquisition strategy to gain the short-term benefits of BNF and the long-term benefits of exoenzyme release.

BNF allows free-living diazotrophs using the LAH N acquisition strategy to gain several concrete advantages when competing with non-diazotrophs over soil N. First, free-living diazotrophs can put more cellular N acquired from LMW N uptake toward growth than non-diazotrophs, whereas this resource is available. As they cannot rely on BNF to offset the cellular N investments necessary for exoenzyme production, non-diazotrophic microbes must divert a portion of the cellular N they acquire through LMW N uptake away from growth and toward exoenzyme production at relatively high LMW N availability (that is, levels of LMW N illustrated in Figure 1b); by budgeting cellular N in this way non-diazotrophs retain the ability to release N-acquiring exoenzymes and do not risk losing direct access to the HMW N pool. As they can access atmospheric N, free-living diazotrophs using the LAH N acquisition strategy can put cellular N obtained through LMW N uptake entirely toward growth without losing the ability to acquire HMW N through exoenzyme release. Furthermore, by supplementing LMW N uptake with BNF, free-living diazotrophs likely maintain higher growth rates than non-diazotrophs during times of low LMW N availability.

Finally, by using the LAH N acquisition strategy, free-living soil diazotrophs should put less cellular N toward HMW N acquisition than non-diazotrophic soil microbes overall. Although non-diazotrophic soil microbes do rely on low LMW N availability to induce exoenzyme production, BNF allows free-living soil diazotrophs to begin exoenzyme production when LMW N is entirely exhausted without losing access to the HMW N pool. These behavioral differences should result in a temporal delay between exoenzyme release by non-diazotrophic soil microbes and free-living soil diazotrophs inhabiting the same environment. However, although they invest less in exoenzyme production, free-living soil diazotrophs will likely acquire similar amounts of N as non-diazotrophic soil microbes as the products of exoenzyme production are available to all microbes in a given environment.

### Evidence for the LAH N acquisition strategy in pure culture

Although the LAH N acquisition strategy has yet to be rigorously tested in pure culture, we present three examples of indirect evidence for its existence in the behavior of diazotrophs. The examples involve (1) ZAS-9, a termite gut spirochete, (2) *Azoarcus sp.* Strain BH72, a plant endophyte that also exists as a rhizosphere free-living diazotroph and (3) *Azotobacter vinelandii*, a model free-living soil diazotroph.

ZAS-9, a termite gut spirochete, was isolated by Lilburn *et al.* (2001) with the intention of testing this organism for BNF. As the organism required an unknown component of yeast extract (YE) for growth, testing was conducted with media containing 1% YE, which contains various fixed N sources including 16.6 mg protein ml^–1^ (Lilburn *et al.*, 2001). Although the N contained in the YE allowed ZAS-9 to grow in an argon atmosphere, Lilburn *et al.* (2001) showed that 30% more growth occurred when ZAS-9 was grown in an N_2_ atmosphere, indicating diazotrophy. The authors confirmed BNF by re-incubating ZAS-9 with an atmosphere of >99.9% ^15^N_2_ gas and measuring the ^15^N content of biomass after incubation. Lilburn *et al.* (2001) expected to see ~38% ^15^N in the biomass of ZAS-9, reflective of the growth difference the organism exhibited in an argon vs N_2_ atmosphere. However, the authors only saw ~6% ^15^N incorporation into biomass.

Based on their isotopic data, Lilburn *et al.* (2001) concluded that BNF allowed ZAS-9 to access additional sources of fixed N from the media. We agree and suggest that ZAS-9 used the LAH strategy we introduce here as follows: During incubation in an argon atmosphere, ZAS-9 used inorganic N available in YE, and then switched to LMW N to meet cellular N demand. However, ZAS-9 could not use atmospheric N to supplement cellular N demand in conjunction with LMW N uptake in the argon atmosphere. As BNF allows free-living diazotrophs to drain LWM N pools below levels necessary to meet cellular N investments for exoenzyme production, ZAS-9 was not able to access HMW N sources in the YE in the argon atmosphere. However, when atmospheric N was available, ZAS-9 used cellular N acquired through BNF to fuel exoenzyme production at low LMW N availabilty. Once exoenzymes accumulated in the medium, the degradation products of HMW N became available to ZAS-9 and the organism decreased reliance on atmospheric N in favor of LMW N sources. The LAH strategy thus explains both the additional growth in the presence of atmospheric N and the low levels of isotopic incorporation observed by Lilburn *et al.* (2001). Although ZAS-9 is not a free-living soil diazotroph, its native environment is characterized by low diffusion and high availability of HMW N that could support exoenzyme production as a means of N acquisition, similar to soil environments.

*Azoarcus* sp. strain BH72 is a diazotroph that lives both as a free-living soil diaoztroph and as an endophyte of grasses (Reinhold-Hurek and Hurek, 2011), colonizing through the root surface (Hurek *et al.*, 1994). Sarkar and Reinhold-Hurek (2014) profiled the transcriptome of this model organism both when supplied with excess NH_3_ and when engaged in BNF. They found that putative genes for type II secretory pathways were upregulated during BNF. The authors noted their surprise at the increased expression of genes associated with protein excretion and therefore used reverse-transcriptase PCR to confirm that *azo0805*, a putative component of the type II excretory pathway, was upregulated ~21-fold during BNF.

Hamilton *et al.* (2011) conducted a similar transcriptional-profiling study to compare the transcriptome of *Azotobacter vinelandii* when engaged in BNF with the transcriptome when the organism was supplied with NH_3_. They found that the largest increases in gene expression during BNF were related to the production type IV pili. The authors suggested that *A. vinelandii* cells may produce type IV pili to aid in aggregation as a mechanism of oxygen protection for oxygen-sensitive nitrogenase proteins. However, Peña *et al.* (2002) found that aggregation in *A. vinelandii* decreases with dissolved oxygen concentration, making this an unlikely explanation. Similar to the findings of Sarkar and Reinhold-Hurek (2014), we suggest that the overexpression of type IV pili genes could indicate increased exoenzyme transport by *A. vinelandii*. Components of the type IV pili biogenesis system share structural similarity with components of type II secretion pathways (Peabody *et al.*, 2003; Yamagata *et al.*, 2012), and organisms have been shown to use type IV pili mechanisms to secrete both chitinases (Han *et al.*, 2007) and proteases (Forsberg and Guina, 2007).

The transcriptional-profiling studies by Sarkar and Reinhold-Hurek (2014) and Hamilton *et al.* (2011) show upregulation of protein secretion genes during BNF by free-living soil diazotrophs. This behavior is consistent with the LAH N acquisition strategy, as these secretion pathways could be used to release N-acquiring exoenzymes, and this behavior occurred at conditions when both inorganic N and LMW N were limiting. However, neither study showed that genes for N-acquiring exoenzymes were upregulated during BNF. This is likely due to the fact that HMW N was not present in the media, as substrate availability is a necessary precondition for the production of N-acquiring exoenzymes through the mechanism of substrate induction (Felse and Panda, 1999; Geissler *et al.*, 2010 and references therein). In soils, HMW N availability is likely never low enough to limit exoenzyme production, so repression by inorganic N and LMW N serve as the main source of regulation. Transcriptional-profiling studies of free-living diazotrophs conducted in the presence of HMW N are necessary to better understand the N acquisition strategies of these organisms in soils.

## Ecosystem-level ramifications of the LAH N acquisition strategy by free-living diazotrophs

Acknowledging that free-living soil diazotrophs secrete N-acquiring exoenzymes changes our understanding of the role that these organisms have in natural systems. Overall, access to the HMW N pool should decrease the proportion of cellular N that free-living soil diazotrophs obtain by BNF regardless of the N acquisition strategies these organisms use in nature. Estimated rates of fixation by free-living soil diazotrophs in terrestrial systems are usually low in comparison with commonly applied rates of fertilizer addition and rates of symbiotic N fixation (Cleveland *et al.*, 1999; Reed *et al.*, 2011) despite the fact that free-living diazotrophs can be detected in a wide variety of unmanaged terrestrial environments (for example, Wang *et al.*, 2013). Together these observations suggest that free-living soil diazotrophs are not heavily reliant on BNF to satisfy their cellular N demand. Furthermore, a recent meta-analysis by Rocca *et al.* (2015) failed to find a significant relationship between BNF and diazotroph abundance (as measured by *nifH* gene abundance); this finding suggests that per-cell fixation rates by free-living soil diazotrophs vary across systems, likely in relation to soil N pools as we suggest here.

Many fertilization studies have been conducted to investigate the role that fixed N has in regulating ecosystem-level BNF. Most often, soils are supplemented with inorganic N, which reliably represses BNF across systems (Reed *et al.*, 2011 and references therein). However, we caution that the form of N used in these studies may markedly affect the results. Fertilization with LMW N may only partially inhibit BNF by a proportion of the diazotrophic community and fertilization with HMW N could actually stimulate BNF by free-living soil diazotrophs using the LAH N acquisition strategy. For example, fertilization with chitin has been shown to increase extracellular chitinase abundance (Johnson-Rollings *et al.*, 2014) and activity (Allison *et al.*, 2014) in soils through the mechanism of substrate induction. Chitinase production by free-living soil diazotrophs is likely stimulated by chitin addition as well, and these organisms are predicted to use BNF to meet cellular N investments necessary for chitinase production if they adhere to the LAH N acquisition strategy we introduce here. The authors of these studies may have seen increased rates of BNF in response to chitin addition had they measured this process as well.

Free-living soil diazotrophs may also affect observed patterns of exoenzyme activity in soils if these organisms make up a substantial proportion of the organisms capable of producing a certain N-acquiring exoenzyme in a given environment. As the LAH N acquisition strategy allows free-living soil diazotrophs to temporally delay exoenzyme production in relation to non-diazotrophic soil microbes, soils with high levels of free-living diazotrophs may have two measurable pulses of exoenzyme production: an early pulse that occurs before LMW N availability limits production by non-diazotrophic soil microbes and a later pulse from exoenzyme release by free-living soil diazotrophs. We have also noted that free-living soil diazotrophs may participate less in the production of N-acquiring exoenzymes than non-diazotrophic soil microbes. The presence of organisms that do not participate in exoenzyme release has the potential to decrease overall levels of exoenzyme production in modeling studies (Allison, 2005), and decreased participation in exoenzyme release by free-living soil diazotrophs could have similar effects.

## Future research

Both pure culture and environmental studies are necessary to improve our understanding of how the production of N-acquiring exoenzymes may affect ecosystem-level BNF by free-living diazotrophs. Pure culture studies should focus on documenting the occurrence of exoenzyme production by free-living diazotrophs and investigating how the presence of HMW N affects BNF by these organisms. Environmental studies should focus on process-level links between BNF and the activity of N-acquiring exoenzymes in natural soils. Examining temporal relationships between these processes could be especially powerful. Rigorous tests of both the general connections between HMW N degradation and BNF as well as the specifics of the LAH N acquisition strategy we introduce here will deepen our understanding of environmental N cycling and ultimately enhance quantitative predictions of N fixation rates in terrestrial systems.

## Figures and Tables

**Table 1:** N-acquiring exoenzyme release by free-living soil diazotroph species described since 2000

*Species described*	*N-acquiring exoenzymes*	*Location*	*Reference*
*Hartmannibacter diazotrophicus*	*N-*acetyl-beta-glucosaminidase, caseinase	Germany	Suarez *et al.*, 2014a
*Cellvibrio diazotrophicus*	*N*-acetyl-beta-glucosaminidase	Germany	Suarez *et al.*, 2014b
*Azoarcus olearius*	Gelatinase	Taiwan	Chen *et al.*, 2013
*Chryseolinea serpens*	*N*-acetyl-beta-glucosaminidase	Germany	Kim *et al.*, 2013
*Bacillus rhizosphaerae*	Gelatinase	India	Madhaiyan *et al.*, 2011
*Paenibacillus sonchi*	Caseinase	China	Hong *et al.*, 2009
*Ideonella azotifigens*	Caseinase, gelatinase	USA	Noar and Buckley, 2009
*Paenibacillus forsythiae*	Caseinase	China	Ma and Chen, 2008
*Sphingomonas azotifigens*	Gelatinase	Japan	Xie and Yokota, 2006
*Paenibacillus brasilensis*	Gelatinase, caseinase	Brazil	von der Weid *et al.*, 2002
*Paenibacillus borealis*	Caseinase	Finland	Elo *et al.*, 2001

Abbreviation: N, nitrogen.

**Table 2:** Genomic evidence for exoenzyme production by free-living soil diazotrophs

*Genome searched*	*NCBI ref. seq.*	*SignalP score*	*Annotation*
*Clostridium acetobutylicum* ATCC 9039	WP_010890750.1	0.550	Secreted metalloprotease
	WP_010965812.1	0.727	Extracellular neutral metalloprotease
	WP_010965813.1	0.618	Extracellular neutral metalloprotease
*Clostridium pasteurianum* ATCC 6013	WP_003442650.1	0.707	Peptidase M1
*Paenibacillus beijingensis* 7188	WP_045671766.1	0.621	Serine protease
*Paenibacillus sabinae* T27	WP_025335152.1	0.587	Peptidase
	WP_025335901.1	0.521	Serine protease
	WP_025336974.1	0.626	Peptidase
*Paenibacillus terrae* HPL-003	WP_014277784.1	0.750	Bacillolysin
	WP_014277785.1	0.814	Bacillolysin
	WP_014277786.1	0.760	Bacillolysin
	WP_014282838.1	0.624	Serine protease

SignalP scores are values between 0 and 1 that indicate the likeliness of the presence of a signal peptide in an amino-acid sequence. SignalP scores >0.42 were used to indicate the presence of a signal peptide in Gram-positive bacteria.

**Table 3:** A comparison of inorganic, LMW, atmospheric and HMW N acquisition by free-living soil diazotrophs

*Nitrogen source*	*Examples*	*Availability in soil*	*Acquisition mechanism*	*Energetic investment required*	*Cellular N investment required*	*Immediate return on investment?*	*Constant investment required?*
Inorganic N	NH_4_^+^, NO_3_^−^	Inconsistent	Uptake	Low to moderate (dep. on form and conc.)	Low	Yes (when available)	Yes (but minimal)
LMW N	Amino acids, N-sugars, oligopeptides	Moderate	Uptake and extracellular deamination	Low to moderate (dep. on form and conc.)	Low to moderate	Yes (when available)	Yes (but minimal)
Atmospheric N	N_2_	High	BNF	Moderate to high (dep. on O_2_ availability)	Moderate	Yes	Yes
HMW N	Proteins, chitin, complex soil organic matter	High	Exoenzyme production	Moderate	High	No	No

Abbreviations: BNF, biological nitrogen fixation; HMW, high-molecular weight; LMW, low-molecular weight; N, nitrogen.
