# Supplementary material for: Complex N acquisition by soil diazotrophs: how the ability to release exoenzymes affects N fixation by terrestrial free-living diazotrophs
Source: ISME J. 2016 Nov 29;11(2):315–26. doi: 10.1038/ismej.2016.127 (PMC5270568; doi:10.1038/ismej.2016.127)
Supplement: Supplementary Table 1 [file ismej2016127x1.pdf]

Supplemental Table 1. Genomic evidence for extracellular deamination by free-living soil diazotrophs. SignalP scores are values between 0 and 1 that indicate the likeliness of the presence of a signal peptide in an amino acid sequence. Signal P scores greater than 0.42 were used to indicate the presence of a signal peptide in gram-positive bacteria. We found no genomic evidence for extracellular deamination by *Clostridium acetobutylicum* (ATCC 9039) or *Clostridium pasteurianum* (ATCC 6013), which are therefore not listed in this table.

**Genome searched**	**NCBI Ref. seq.**	**SignalP score**	**Annotation**
Paenibacillus beijingensis 7188	WP_045670562.1	0.871	copper amine oxidase
	WP_045671836.1	0.543	copper amine oxidase
	WP_045672389.1	0.689	copper amine oxidase
	WP_045673106.1	0.710	copper amine oxidase
Paenibacillus sabinae T27	WP_025333104.1	0.771	copper amine oxidase
	WP_025334629.1	0.625	copper amine oxidase
	WP_025334738.1	0.764	copper amine oxidase
Paenibacillus terrae HPL-003	WP_014278449.1	0.688	copper amine oxidase
	WP_014278450.1	0.438	copper amine oxidase
	WP_014278514.1	0.567	copper amine oxidase
	WP_014278677.1	0.704	copper amine oxidase
	WP_014279290.1	0.720	copper amine oxidase
	WP_014279946.1	0.858	copper amine oxidase
	WP_014280075.1	0.685	copper amine oxidase
	WP_014280130.1	0.432	copper amine oxidase
	WP_014280165.1	0.616	copper amine oxidase
	WP_014280193.1	0.787	copper amine oxidase
	WP_014280782.1	0.672	copper amine oxidase
	WP_014281017.1	0.724	copper amine oxidase
	WP_014282346.1	0.651	copper amine oxidase
	WP_014282566.1	0.557	copper amine oxidase
	WP_014282725.1	0.698	copper amine oxidase
	WP_043922553.1	0.829	copper amine oxidase
	WP_043922806.1	0.799	copper amine oxidase
